# Toward criteria for pragmatic measurement in implementation research and practice: a stakeholder-driven approach using concept mapping

**DOI:** 10.1186/s13012-017-0649-x

**Published:** 2017-10-03

**Authors:** Byron J. Powell, Cameo F. Stanick, Heather M. Halko, Caitlin N. Dorsey, Bryan J. Weiner, Melanie A. Barwick, Laura J. Damschroder, Michel Wensing, Luke Wolfenden, Cara C. Lewis

**Affiliations:** 1 Department of Health Policy and Management, Gillings School of Global Public Health, University of North Carolina at Chapel Hill, 1105C McGavran-Greenberg Hall, 135 Dauer Drive, Campus Box 7411, Chapel Hill, NC 27599 USA; 2 Hathaway-Sycamores Child and Family Services, Pasadena, CA USA; 3 Department of Psychology, University of Montana, Missoula, MT USA; 4 Kaiser Permanente Washington Health Research Institute, Seattle, WA USA; 5 Department of Global Health and Department of Health Services, University of Washington, Seattle, WA USA; 6 Hospital for Sick Children, University of Toronto, Toronto, ON Canada; 7 VA Ann Arbor Center for Clinical Management Research and Diabetes QUERI, VA Ann Arbor Healthcare System, Ann Arbor, MI USA; 8 Department of General Practice and Health Services Research, University Hospital Heidelberg, Im Neuenheimer Feld, Heidelberg, Germany; 9 School of Medicine and Public Health, The University of Newcastle, Callaghan, NSW Australia

## Abstract

**Background:**

Advancing implementation research and practice requires valid and reliable measures of implementation determinants, mechanisms, processes, strategies, and outcomes. However, researchers and implementation stakeholders are unlikely to use measures if they are not also pragmatic. The purpose of this study was to establish a stakeholder-driven conceptualization of the domains that comprise the pragmatic measure construct. It built upon a systematic review of the literature and semi-structured stakeholder interviews that generated 47 criteria for pragmatic measures, and aimed to further refine that set of criteria by identifying conceptually distinct categories of the pragmatic measure construct and providing quantitative ratings of the criteria’s clarity and importance.

**Methods:**

Twenty-four stakeholders with expertise in implementation practice completed a concept mapping activity wherein they organized the initial list of 47 criteria into conceptually distinct categories and rated their clarity and importance. Multidimensional scaling, hierarchical cluster analysis, and descriptive statistics were used to analyze the data.

**Findings:**

The 47 criteria were meaningfully grouped into four distinct categories: (1) acceptable, (2) compatible, (3) easy, and (4) useful. Average ratings of clarity and importance at the category and individual criteria level will be presented.

**Conclusions:**

This study advances the field of implementation science and practice by providing clear and conceptually distinct domains of the pragmatic measure construct. Next steps will include a Delphi process to develop consensus on the most important criteria and the development of quantifiable pragmatic rating criteria that can be used to assess measures.

## Background

Bridging the gap between research and practice by advancing implementation science will require valid and reliable measures of implementation determinants, mechanisms, processes, strategies, and outcomes [1]. However, implementation stakeholders (i.e., researchers and practice-based implementers) are unlikely to use measures solely on the basis of strong psychometric properties; they also need to be pragmatic [2, 3]. For example, a measure that is psychometrically sound, but is time-consuming or expensive to administer, is unlikely to be used. There is currently no consensus about what constitutes a pragmatic measure. Glasgow and Riley [2] advanced the conceptualization of the pragmatic measure construct by suggesting two types of criteria: required (important to stakeholders, low burden for respondents and staff, actionable, and sensitive to change) and recommended (broadly applicable, used for benchmarking, unlikely to cause harm, psychometrically strong, and related to theory or model). However, these recommendations may be limited as they were not developed through a systematic literature review, were not informed by relevant stakeholders, and focused on clinical measures. Key aspects of the pragmatic measure construct may have been overlooked.

The “Advancing Implementation Science through Measure Development and Evaluation” study [3] aims to (1) establish a stakeholder-driven operationalization of pragmatic measures and develop reliable, valid rating criteria for assessing this construct; (2) develop reliable, valid, and pragmatic measures of three different implementation outcomes [4] (acceptability, appropriateness, and feasibility) [5]; and (3) identify measures that demonstrate both psychometric and pragmatic strength. This article details our Aim 1 efforts to establish a stakeholder-driven conceptualization of the domains that comprise the pragmatic measure construct. As a first step toward that aim, we conducted a systematic review of the literature and semi-structured interviews with stakeholders drawn from multiple organization types (e.g., community mental health center, school-based mental health, state mental health department, residential treatment center, and inpatient hospital) and service roles (e.g., administrators and clinicians). The eight relevant articles from the systematic review and the seven semi-structured interviews ultimately yielded 47 potential criteria for pragmatic measures (e.g., low cost, efficient, easy to score) after duplicates were removed [6].

The present study engaged stakeholders with experience implementing behavioral health interventions in a concept mapping activity [7] to explore the relationships between the criteria, to develop conceptually distinct categories, and to assess the clarity and importance of the criteria. Considering this list of criteria and the associated ratings of clarity and importance will help implementation stakeholders to develop or select measures that are pragmatic. The findings of this study will assist us in refining and consolidating the list of criteria as we work toward developing a valid and reliable set of rating criteria for assessing the extent to which a measure is pragmatic. Ultimately, the rating criteria will be applied to implementation-related measures of constructs associated with the Consolidated Framework for Implementation Research [8] and the Implementation Outcomes Framework [4] in Aim 3 of our study [3].

## Methods

### Participants

Purposeful sampling [9] was used to recruit stakeholders (*N* = 24) with experience implementing behavioral health interventions and to ensure maximum variation in discipline, setting, and geographic location. The stakeholders were administrators (*n* = 13), clinicians (*n* = 6), and researchers (*n* = 5) with an average of 10 (SD = 9) years of implementation experience. They worked in community mental health (*n* = 10); specialty mental health, outpatient mental health, or private practice (*n* = 3); community organizations (*n* = 3); primary care (*n* = 2); children’s social services (*n* = 1); inpatient psychiatry (*n* = 1); schools (*n* = 1); government agencies (*n* = 1); and other settings (*n* = 2). Twenty-four stakeholders are above the recommended sample size for concept mapping (≥ 15) [10].

### Data collection

Stakeholders completed a concept mapping activity, which is a structured process designed to organize concepts into categories and generate ratings of specified dimensions [7, 11, 12]. It is particularly useful for structuring the ideas of diverse groups of stakeholders and has been used in implementation research for multiple purposes, including identifying and prioritizing implementation barriers and facilitators [13, 14], organizing implementation strategies [15], and identifying training needs [16]. Concept mapping is an inherently mixed methods approach that involves multiple steps, typically including brainstorming, statement analysis and synthesis, unstructured sorting of statements, multidimensional scaling and cluster analysis, and the generation of interpretable maps and data displays [7]. Thorough and accessible introductions to the concept mapping method can be found in Trochim and Kane [11] and Kane and Trochim [7].

The criteria for pragmatic measures were generated through a systematic review of the literature and semi-structured interviews with stakeholders (described above) that yielded 47 criteria after duplicates were removed [6]. The Concept Systems Global MAX™ [17] web-based platform was used to collect and analyze the data for this study asynchronously. After logging on to the web-based platform, participants were asked to complete basic demographic questions (primary role, work setting, years of experience, and race/ethnicity). They were then asked to complete an unstructured sorting task that involved sorting each of the 47 criteria into conceptually similar groups and giving each category a name that describes its theme or contents. They were instructed not to sort based upon priority or value (e.g., “important” or “hard to do”) or to create “miscellaneous” or “other” piles that grouped dissimilar items. It was also noted that the number of categories participants create typically varies from 5 to 20; however, there was no mandate to stay within that range. To help us to determine the criteria that may be most helpful as we move toward developing concrete rating scales for the pragmatic construct, we asked stakeholders to rate each criterion’s clarity and importance on a 10-point scale (1 = not at all clear/not at all important, 10 = incredibly clear/incredibly important). Data collection was completed in 2 months.

### Data analysis

Through the Concept Systems Global MAX™ [17] web-based platform, multidimensional scaling and hierarchical cluster analysis were used to generate visualizations of the relationships between the pragmatic criteria. Multidimensional scaling was used to generate a point map depicting each of the pragmatic criteria and relationships between them based upon a summed square similarity matrix [11]. Criteria frequently sorted together were placed closer together on the point map. Hierarchical cluster analysis was used to partition the point map into non-overlapping clusters [11].

The analytic process involved the investigative team considering a range of cluster solutions, deciding which solution best suited the purposes of the current study, and labeling each cluster [7, 11]. Concept Systems Global MAX™ [17] aids in the labeling process by suggesting potential cluster labels based upon participant responses. These labels do not always adequately reflect the items within a cluster; however, in at least one case, we used a variant of the suggested label, and in others, the suggested labels inspired us to generate labels that had similar meanings as we sought to obtain consensus among the investigative team. In two cases, individual items were moved from one cluster to another to improve the clarity and consistency of the clusters [7]. Model fit was assessed using the stress value, an indicator of goodness of fit between the point map and the total similarity matrix. Cross-study syntheses of concept mapping studies have consistently found mean stress values of 0.28 [7, 10, 12], with higher stress scores indicating poorer representation of the data. The final cluster solution and associated labels were vetted by a stakeholder panel that included four of the seven individuals (three did not respond) from the semi-structured interview study [6] and by all nine members of the parent study’s International Advisory Board, which is comprised of leading implementation scientists purposefully selected to represent broad expertise and geographic diversity. The four stakeholders participated in both the interview study and the concept mapping exercise, while members of the International Advisory Board were not involved in either study as participants.

Descriptive statistics were used to characterize clarity and importance ratings at both the criterion and cluster levels. At the criterion level, means for both clarity and importance are captured in Table 1 and in Fig. 3 which depicts a “go zone” graph [7, 11]. The go zone graph is a bivariate plot of the clarity and importance ratings that is divided into quadrants based upon the mean values of those dimensions. Thus, criteria that fall into quadrant I are above the mean for both importance and clarity, whereas criteria that fall into quadrant III are below the mean for both importance and clarity. The go zone quadrants for each criterion are listed in Table 1 as well to ease the interpretation of each criterion’s mean ratings. Finally, the overall mean for importance and clarity was calculated for each cluster and is shown in a ladder graph (or pattern match) [7, 11] in Fig. 2.

**Table 1:** Mean clarity and importance ratings for each criterion (*n* = 24)

#	Criterion	Clarity	Importance	Quad.
Acceptable				
4	Creates a low social desirability bias	5.21	5.88	III
22	Transparent	6.75	6.92	III
24	Acceptable (to staff and clients)	7.83	8.50	I
25	Tied to reimbursement	8.00	5.08	II
28	Relevant	7.21	8.71	IV
30	Offers relative advantage over ex	7.33	7.54	IV
43	Low cost	8.67	8.04	I
Compatible				
3	Applicable	7.25	8.25	IV
8	Efficient	7.79	8.21	I
12	Focused	5.92	6.92	III
16	The output of routine activities	6.58	7.21	III
37	Not used for staff punishment	7.63	7.63	I
40	Non-duplicative	7.21	7.50	III
Easy				
9	Offers flexible administration time	6.88	6.92	III
10	Easy to interpret	8.88	8.38	I
15	Creates low assessor burden (ease of training, scoring, administration time)	8.50	7.75	I
17	Easy to administer	8.75	8.13	I
20	Not wordy	8.79	6.38	II
21	Completed with ease	8.75	7.71	I
23	Requires no expertise	7.46	4.75	III
26	Of low complexity	7.58	6.42	III
27	Uses accessible language	7.75	8.13	I
31	Accessible by phone	8.29	4.88	II
32	Brief	8.21	6.92	II
34	Intuitive	6.29	6.25	III
36	Feasible	7.00	8.25	IV
39	Simple	7.54	7.17	III
41	Easy to use	8.29	8.00	I
42	Easy to score	8.88	7.75	I
44	One that offers automated scoring or can be scored elsewhere	8.63	6.71	II
45	Offers a compatible format to setting/user	5.63	7.29	III
47	Low burden	7.33	8.21	IV
Useful				
1	Informs decision making	8.00	8.71	I
2	Fits organizational activities	8.21	7.96	I
5	Provides a cut-off score leading to an intervention or treatment plan	7.63	6.96	II
6	Connects to clinical outcomes	8.38	8.83	I
7	Important to clinical care	7.96	8.92	I
11	Produces reliable and valid results	9.13	9.25	I
13	Reveals problems/issues in process or outcomes	6.79	6.67	IV
14	Informs adherence of fidelity	7.54	7.42	III
18	Assesses organizational progress over time	7.50	7.67	IV
19	Sensitive to change	7.25	7.25	III
29	Meaningful	6.79	8.71	IV
33	Confirms efficacy of interventions	7.13	7.92	IV
35	Has a meaningful score distribution	6.54	6.71	III
38	Optimizes patient care	7.46	8.83	IV
46	Informs clinical intervention selection	7.92	8.29	I

## Results

Valid sorts were obtained from 23 stakeholders. One stakeholder sorted the criteria into value-based categories (e.g., “not that important”) and was dropped from the multidimensional scaling and hierarchical cluster analyses. All 24 stakeholders provided valid ratings of clarity and importance.

**Fig. 1:**
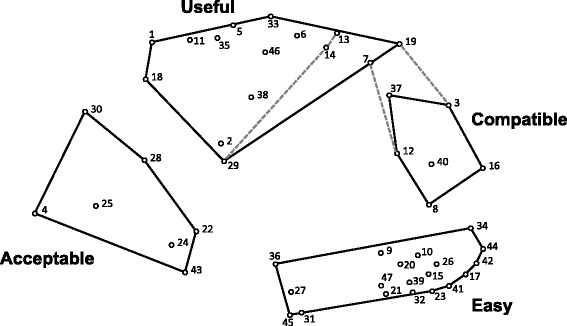
Point and cluster map of criteria demonstrating spatial relationships (*n* = 23). This point and cluster map reflects the product of our stakeholders’ (valid response *n* = 23) sorting the 47 criteria into groups that they deemed conceptually similar. Each strategy is depicted as a dot with a number that corresponds to Table 1. The distances between criteria reflect the frequency at which they were sorted together; thus, strategies that were sorted together frequently are closer together on the map. These spatial relationships are relative to the data in this study and do not reflect an absolute relationship (i.e., a 5-mm distance on this map does not reflect the same relationship as a 5-mm distance on a map from a different dataset) [15]. Items 19 (“sensitive to change”) and 7 (“important to clinical care”) were originally assigned to the “compatible” cluster, but were moved to the “useful” cluster because the investigative team believed that it represented a better conceptual fit. The gray dotted lines within the “useful” cluster and between the “useful” and “compatible” clusters represent how the clusters would have been represented if we had not made this change

The investigative team independently considered cluster solutions ranging from ten to two clusters and came to consensus on a four-cluster solution with the following labels: acceptable, compatible, easy, and useful. The stakeholder panel (*n* = 4) and an International Advisory Board (*n* = 9) vetted the four-cluster solution and agreed that it balanced parsimony and distinction between the domains. The stress value was 0.2581, demonstrating goodness of fit [7, 10, 12]. Figure 1 shows the final point and cluster map that visually represents the relationships between the 47 criteria. Each point in Fig. 1 represents a single criterion, which are labeled numerically to facilitate cross-referencing the point and cluster map with the full descriptions listed in Table 1. Two criteria (“important to clinical care” [#7] and “sensitive to change” [#19]) were moved from the “Compatible” cluster to the “Useful” cluster to enhance conceptual clarity and consistency.

**Fig. 2:**
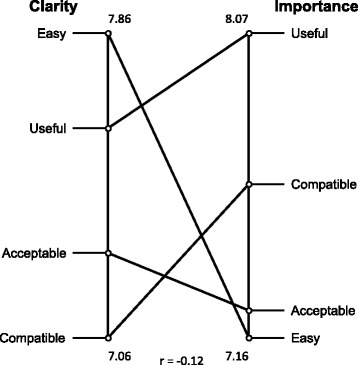
Mean clarity and importance ratings per cluster (*n* = 24)

**Fig. 3:**
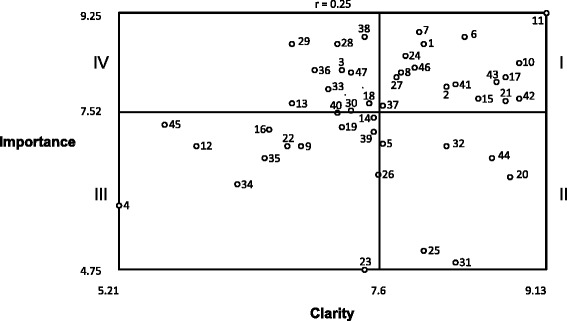
Go-zone graph of mean clarity and importance ratings (*n* = 24). The range of the *x-* and *y-*axes reflect the mean values obtained for all 47 of the pragmatic criteria for the clarity and importance rating scales. The plot is divided into quadrants based upon the overall mean values for each rating scale: *quadrant I* (above the mean for both clarity and importance), *quadrant II* (above the mean for clarity, below the mean for importance), *quadrant III* (below the mean for clarity and importance), and *quadrant IV* (below the mean for clarity, above the mean for importance)

Figure 2 shows the mean clarity and importance ratings at the cluster level. Overall, the mean ratings for the clusters were relatively high for both clarity (7.06–7.86) and importance (7.16–8.06). Figure 3 depicts a “go-zone” graph that shows clarity and importance ratings for each of the 47 criteria and where each criterion falls with respect to the overall mean rating for clarity (7.6) and importance (7.52).

## Discussion

To usefully inform the assessment of implementation determinants, mechanisms, processes, strategies, and outcomes, measures must be both psychometrically sound and pragmatic. This study advances previous work [2, 6] by engaging stakeholders to conceptualize domains that comprise the pragmatic measure construct. The 47 criteria previously identified through a systematic literature review and semi-structured interviews [6] were grouped into four categories: acceptable, compatible, easy, and useful. The overarching categories should be helpful in considering the pragmatic construct and have the advantage of parsimony. However, at this stage of development, we suggest that readers consider the nuances of the pragmatic construct that are represented at the criterion level.

Ratings of clarity and importance at criterion and cluster levels were generally high. Implementation stakeholders interested in using these criteria to inform the development or assessment of measures may wish to focus on the criteria that fell within the go zone (i.e., above the overall mean for both importance and clarity), as those criteria are likely closer to being useable in their current form. Ratings for some of the other criteria suggested items that need to be removed due to lack of importance (e.g., “requires no expertise,”) or edited due to lack of clarity (e.g., “focused”).

This study is a step toward developing rating criteria that could inform measure development and the assessment of measures’ pragmatic qualities, which ultimately will benefit research and practice by yielding and revealing measures that are psychometrically strong *and* pragmatic, possibly increasing their future use. Next steps will include developing consensus on the relative priorities for these categories and criteria through a Delphi [18] study; developing rating criteria with concrete, measurable anchors; and assessing inter-rater reliability and known-groups validity of the criteria [3]. Longer-term objectives are to combine the pragmatic rating criteria with evidence-based rating criteria and apply both to a repository of over 450 measures to assess their psychometric and pragmatic strength [3, 19]. The resulting pragmatic rating scale may also influence reporting guidelines for implementation measures and measure development procedures.

Several limitations should be noted. First, it is possible that engaging our 24 stakeholders in an open process of brainstorming could have yielded a more comprehensive list of potential criteria for the pragmatic construct. Our use of both a systematic literature review and semi-structured interviews with key stakeholders to identify dimensions of the pragmatic construct should largely assuage this concern. Second, our sample primarily included US-based stakeholders working in behavioral health. It is possible that a more diverse group would sort and rate these criteria differently. However, to ensure the relevance of these findings to international stakeholders, we sought input regarding the interpretation and presentation of these findings from our International Advisory Board and learned that the categories and criteria resonated with them. Third, our sample included administrators, clinicians, and researchers; however, it did not include policy makers, who may have rated these criteria differently. Including a larger sample with more diverse stakeholders would have allowed us to examine whether ratings of importance and clarity differed based upon role or work setting, which Aarons et al. [13] have found to be the case in a concept mapping study of stakeholders’ perceptions of implementation barriers and facilitators. We believe that these criteria should be generalizable to other contexts, but as they are further developed and applied, it will be important to examine whether they are readily applicable to a diverse array of stakeholders and contexts. Finally, while concept mapping provides a rigorous, mixed methods approach to engaging diverse stakeholders and generating conceptual clarity, there are cases in which the way individual items are grouped does not exactly fit with one’s intuitive sense of where they might belong. In some cases when items are located adjacent to a cluster that may provide a better fit, these items can be re-assigned as we have done with two of the criteria in this study. In other cases, it is not empirically justified to reassign items. These decisions reflect the judgment of the investigative team and other stakeholders, and others may consider these items differently.

## Conclusions

This study provides a preliminary list of stakeholder-driven criteria for evaluating the pragmatic qualities of implementation measures. The categories and ratings of these criteria assist in further refinement of the pragmatic construct and facilitate efforts to immediately apply the criteria that appear to be the most clear and important. Ultimately, we hope this nudges the field toward the use of measures that are valid, reliable, *and* pragmatic.
